# Current Advances in PD-1/PD-L1 Blockade in Recurrent Epithelial Ovarian Cancer

**DOI:** 10.3389/fimmu.2022.901772

**Published:** 2022-06-27

**Authors:** Yuedi Zhang, Qiulin Cui, Manman Xu, Duo Liu, Shuzhong Yao, Ming Chen

**Affiliations:** ^1^ Department of Gynecology, The First Affiliated Hospital of Sun Yat-sen University, Guangzhou, China; ^2^ School of Medicine, Sun Yat-sen University, Guangzhou, China

**Keywords:** programmed death-1 (PD-1), programmed death ligand 1 (PD-L1), immunotherapy, recurrent ovarian cancer, cold tumor

## Abstract

Immunotherapies have revolutionized the treatment of a variety of cancers. Epithelial ovarian cancer is the most lethal gynecologic malignancy, and the rate of advanced tumor progression or recurrence is as high as 80%. Current salvage strategies for patients with recurrent ovarian cancer are rarely curative. Recurrent ovarian cancer is a “cold tumor”, predominantly due to a lack of tumor antigens and an immunosuppressive tumor microenvironment. In trials testing programmed death-1 (PD-1)/programmed death ligand 1 (PD-L1) blockade as a monotherapy, the response rate was only 8.0-22.2%. In this review, we illustrate the status of cold tumors in ovarian cancer and summarize the existing clinical trials investigating PD-1/PD-L1 blockade in recurrent ovarian cancer. Increasing numbers of immunotherapy combination trials have been set up to improve the response rate of EOC. The current preclinical and clinical development of immunotherapy combination therapy to convert an immune cold tumor into a hot tumor and their underlying mechanisms are also reviewed. The combination of anti-PD-1/PD-L1 with other immunomodulatory drugs or therapies, such as chemotherapy, antiangiogenic therapies, poly (ADP-ribose) polymerase inhibitors, adoptive cell therapy, and oncolytic therapy, could be beneficial. Further efforts are merited to transfer these results to a broader clinical application.

## Introduction

Epithelial ovarian cancer (EOC) is a common gynecological cancer and the most lethal gynecological malignancy among women (1). Most patients are diagnosed with advanced stages of this disease (stage III-IV), and the rate of tumor progression or recurrence is as high as 80% (2). The standard treatment for EOC consists of primary debulking surgery (PDS) and platinum plus taxane chemotherapy. Patients with apparently unresectable tumors by imaging study or laparoscopic inspection or patients with low performance status (PS) or medical complications often receive neoadjuvant chemotherapy (NACT), followed by interval debulking surgery (IDS) and subsequent chemotherapy as an alternative choice (3). However, the recurrence of advanced EOC is as high as 70%, with very short overall survival (OS) (4, 5). Therefore, current strategies for recurrent EOC are rarely curative. New treatment strategies are in demand (6, 7). Immunotherapy will be an attractive treatment in the future.

**Figure 1:**
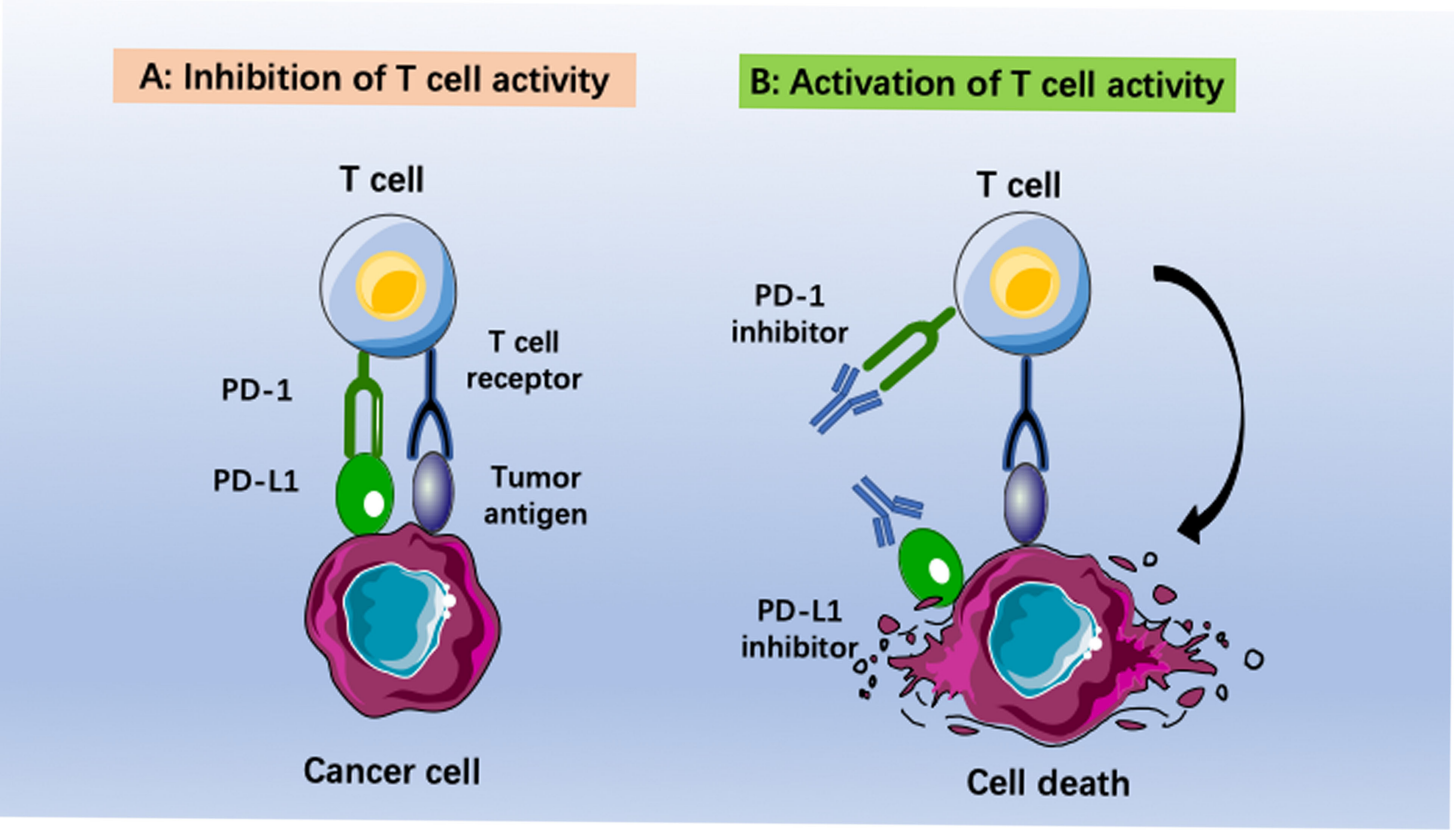
Immunotherapies based on anti-PD-1/PD-L1 pathway antibodies. **(A)** Inhibition of T cell activity caused by binding programmed death ligand 1 (PD-L1) to programmed death (PD-1). **(B)** Activization of T cell activity by using anti-PD-1 or anti-PD-L1 antibodies. The cancer cells become immunogenic again. This leads to recognition of tumor cells by T cells and final elimination by the host immune system.

Checkpoint inhibitor immunotherapy (CPI) has undergone impressive development in recent years. Patients with melanoma, bladder cancer, non-small-cell lung cancer, kidney cancer and colon cancer have reached remarkable long-term survival *via* immunotherapy (8). Among the different types of cancer immunotherapy, immune checkpoint blockade has had the most promising impact. Several antibodies have been approved for therapeutically targeting the programmed cell death 1 (PD1)–PD1 ligand 1 (PD-​L1) axis, T-cell immunoglobulin mucin-3 (TIM-3), or cytotoxic T lymphocyte (CTL)-associated antigen-4 (CTLA-4) (9, 10). Moreover, a large number of monoclonal antibodies (MABs) or small molecules targeting other putative immune checkpoints (such as LAG3, TIGIT, B7H3, CD39, CD73 and adenosine A2A receptor), disrupting negative regulation between tumor cells and T cells, or myeloid cells and T cells, are in clinical and preclinical development (11). Among them, anti-PD-1/PD-L1 antibodies have been the most well studied and evaluated. PD-L1 (B7-H1, CD274) is a 290 aa type I transmembrane protein encoded by the Cd274 gene on mouse chromosome 19 and human chromosome (12). Blocking the interaction between PD-1 and PD-L1 could reverse and/or prevent the exhaustion of tumor-specific T lymphocytes, promoting the surveillance and destruction of tumor cells (**Figure 1**). Many studies have revealed that recurrent EOC is a “cold tumor” (13) that always has an immunosuppressive tumor microenvironment (TME) with few tumor-associated antigens (TAAs) or tumor-specific antigens (TSAs, or neoantigens), resulting in insufficient recognition and eradication of cancer by the immune system. Therefore, only a small subpopulation of EOC patients benefit from PD-1/PD-L1 blockade (14). There is a great demand to improve the efficacy of PD-1/PD-L1 inhibitors in recurrent EOC patients.

In this review, we briefly introduce the characteristics of the TME in EOC and then provide an update of the results of clinical trials of PD-1/PD-L1 blockade therapy in recurrent EOC. Furthermore, the advancement of therapeutic strategies to turn cold tumors into hot tumors will be discussed.

## Literature Review

**Figure 2:**
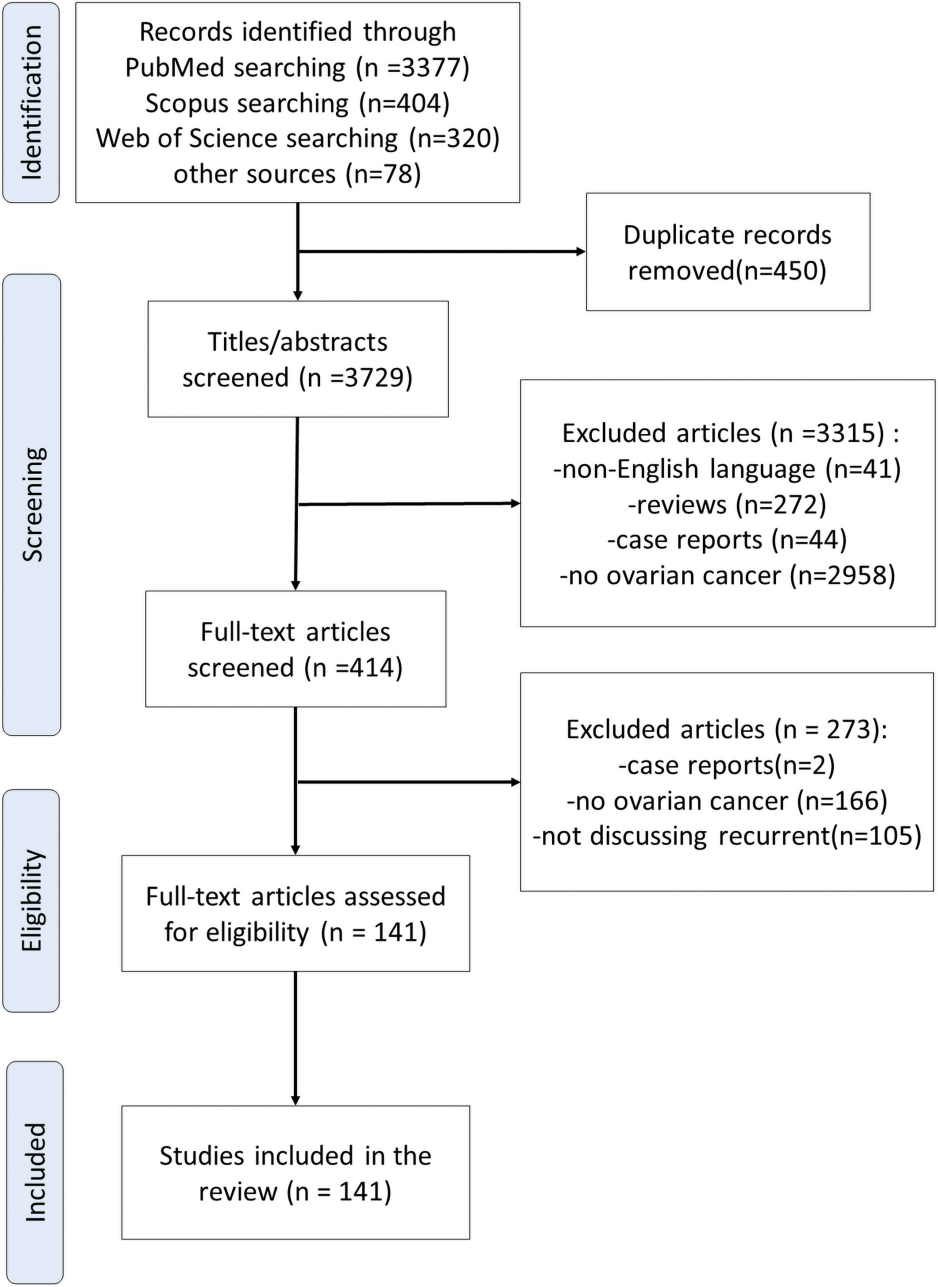
Flow diagram of reference identification and selection.

A comprehensive literature review was conducted using PubMed, Scopus, Web of Science and other sources to identify articles exploring the development of immunotherapy in recurrent EOC, according to the published guidance on narrative reviews (15). We used search terms of “immunotherapy”, “gynecological cancer”, “recurrent ovarian cancer”, “refractory ovarian cancer”, “PD-1/PD-L1 inhibitor”, and “blockade” in different combinations. Search terms were used as key words and as MeSH terms to maximize the output from the literature. Only available full-text articles in English published until September 2021 were included. Additional exclusion criteria were studies on case reports, commentaries and studies not concerning PD-1/PD-L1 inhibitors. The reference lists of the selected articles were reviewed to identify additional articles meeting the eligibility criteria. The database search resulted in 4,179 articles. Finally, after assessing the full-text articles for eligibility and screening of the reference lists, a total of 141 full-text articles were included in the present review (**Figure 2**).

## Cold Tumors: TME of EOC

EOC is classified as a cold tumor and is one of the most difficult malignancies to treat, with an extremely high recurrence rate and a low survival rate. EOC is categorized into four phases based on the International Federation of Gynecology and Obstetrics (FIGO) staging criteria. Early-stage (FIGO stage I-II) EOC can be successfully cured by conventional therapies, including surgery and chemotherapy. In contrast, most advanced EOC patients die predominantly due to relapse and chemoresistance (16). Immunotherapy has opened a new era of cancer treatment, achieving satisfactory efficacy across many tumor types. However, immunotherapy for recurrent EOC has not been approved by the US Food and Drug Administration (FDA) due to the lower efficacy of immunotherapy for this cold tumor. The reason mainly lies in two aspects: 1) a lack of tumor antigens and 2) an immunosuppressive TME.

Tumor antigens have two primary categories: TAAs (relatively restricted to tumor cells) and TSAs (unique to tumor cells) (17). TAAs are antigens present on tumor cells at elevated levels but are also expressed on normal cells at a lower level. TAA peptides fused with human leukocyte antigens (HLAs) can be identified by T somatic cells. In EOC, more than thirty TAAs have been reported to date (18, 19). Research has shown that in the epithelial tissues of the respiratory and reproductive organs, ovarian TAAs are weakly expressed, but in tumor cells, they are always overexpressed (20). CA125 and human epididymis protein 4 (HE4) are two typical TAAs in ovarian tumors, although the diagnostic discrimination value of these markers remains suboptimal due to low sensitivity for early-stage disease (21). In contrast, TSAs, also called tumor neoantigens, can be presented by cancer cells. They contain mutated amino acid sequences generated from genomic perturbations. Such accumulation and preservation of mutations is an important mechanism for cancer development. These mutant peptides presented by HLA class I or class II molecules can prime T cells. However, the mutational burden and the potential of neoantigen expression differ according to cancer types. EOC has a lower tumor mutational burden (TMB) with a mean value of 5.3 mutations/Mb, while the TMB-high category is defined as >10 mutations/Mb. A lack of both TSA and TAA indicates the poor immunogenicity of ovarian tumors. Moreover, a solid tumor comprises heterogeneous populations of tumor cells, in which cancer stem cells (CSCs) can further hamper antigen presentation by reducing the expression of HLA class I molecules (22, 23). CSCs have more potential to escape cytotoxic T-cell killing than other tumor cell populations, providing another resistance against immunotherapy for EOC.

**Figure 3:**
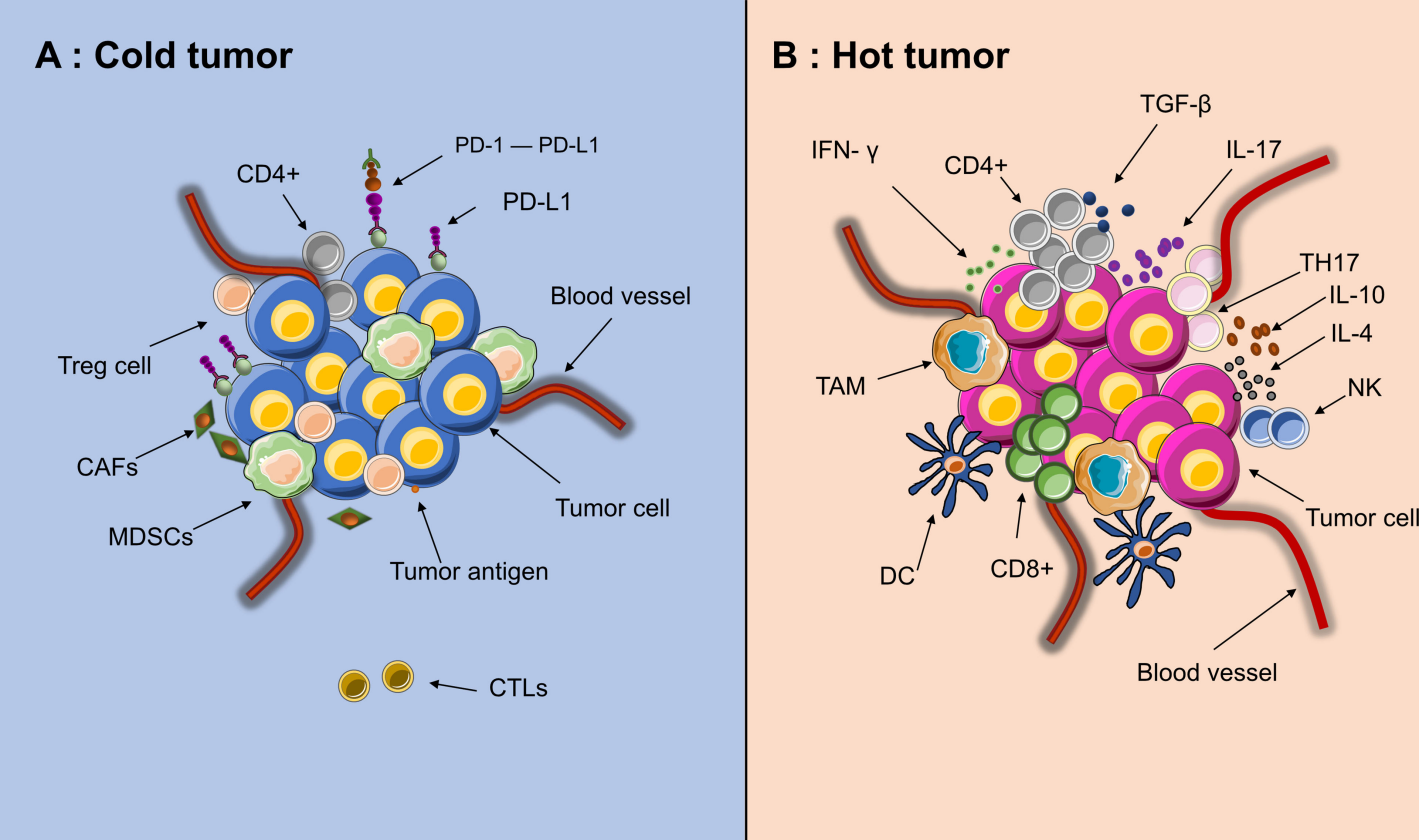
Tumor microenvironment of “hot” and “cold” cancer. **(A)** High activities of effector immune cells, such as CD8+ effector T cells, tumor-associated macrophages (TAM), dendritic cells (DC), IL+17 T cells (TH17) and CD4+ activated T cells. **(B)** High activities of Myeloid-derived suppressor cells (MDSCs), Tregs and CAFs, low activities of CTLs(CD8+)and few recruitment of dendritic cells(DCs). Since the edge of the tumor is in a state of chronic hypoxia, immune cells could migrate from the edge toward the center of tumor, making the core of tumor immunologically hot.

In addition to poor immunogenicity, an immunosuppressive TME also contributes to immunotherapy resistance. Cold tumors have a noninflamed TME. Its characteristics can be summarized as low infiltration of CD8+ T cells and CD4+ T cells as well as increased expression of regulatory T cells (Tregs) and PD-L1+ myeloid cells (24, 25). In cold tumors, immunosuppressive cells, including cancer-associated fibroblasts (CAFs), Tregs and myeloid-derived suppressor cells (MDSCs), are highly active, while the immune response executors CTLs are inactive (**Figure 3**) (26, 27). The lack of effector T cells on a neoplasm leads to a limited patient response to immunotherapy (28). Due to being trapped in the stroma of the tumor or within the peritumoral tissue, CTLs cannot reach the central area (29). Moreover, CAFs also prohibit the immersion of CTLs into the neoplasm and promote a cumulative process of Treg and MDSC into tumors at the center of cancer (30). This status also inhibits the recruitment of dendritic cells (DCs) (31). Furthermore, multiple cell populations, such as MDSCs, macrophages and CSCs, secrete immunosuppressive cytokines, such as transforming growth factor-β (TGF-β), interleukin (IL)-10 and IL-4. These factors can inhibit T-/natural killer (NK) cell activation (32, 33). Nonetheless, a number of abnormalities inside the solid tumor, including abnormal vessels, hypoxia, and an acidic pH, can modulate the interactions among tumor cells, stromal cells, and immune cells and further suppress the immune environment during cancer initiation, progression, and metastasis (34).

Based on the presence or absence of tumor-infiltrating lymphocytes (TIL) and PD-L1 expression in the TME, one immune response classification into four groups has been proposed. The stratification includes type I (PD-L1 positive with TILs driving adaptive immune resistance), type II (PD-L1 negative with no TIL indicating immune ignorance), type III (PD-L1 positive with no TIL indicating intrinsic induction), and type IV (PD-L1 negative with TIL indicating the role of other suppressors in promoting immune tolerance) (28, 35). In advanced melanoma, for which immunotherapy data are the most mature, approximately 38% of patients present with a type I tumor microenvironment and are considered the group that largely benefits from single-agent anti–PD-1/L1 blockade (36). Webb et al. adopted this classification in various histotypes of EOC and found it an important determinant of clinical response (**Table 1**) (37). In HGSC, the Type I pattern (PD-L1^+^/CD8^+^) was most common, followed by the Type IV pattern and the Type II and III patterns. In contrast, the Type IV pattern was more common in the other histological subtypes of EOC. Among all the patients who received standard chemotherapy, the Type I cases of HGSC had the best prognosis (HR 0.2191), suggesting that the immunological benefits associated with adaptive resistance could extend to conventional treatments as well. Moreover, the status of TIL infiltration and PD-L1 expression, or the transition between different types, could be modulated by treatments, such as chemotherapy. Hao et al. calculated an immunological score based on the transcriptomics of 2203 advanced EOC samples from a public dataset. The immune score system selected 69 marker genes and 7 antigen-presenting genes for specific immune cell subtypes. Increased immune scores and cytotoxic activity were found in postchemotherapy tumors compared with their correlated prechemotherapy samples. Patients with high immune scores had a better survival and response to immunotherapy than those with low scores (38). Therefore, the conversion of cold tumors to hot tumors is feasible if EOC has been properly treated. The combined use of immunotherapies and other treatments is promising.

**Table 1:** The immune subtype distribution in different histotypes of ovarian cancer [based on Webb et al. (37)].

Histotype	Percentage of different tumor microenvironment type (%)			
	Type I: Adaptive immune resistance	Type II: Immunological ignorance	Type III: Intrinsic induction	Type IV: Tolerance
	TIL &PD-L1 (+)	TIL &PD-L1 (-)	TIL (-) & PD-L1 (+)	TIL (+) & PD-L1 (-)
High grade serous cancer	57.4	5.1	0	37.4
Low grade serous cancer	0	9.1	0	90.9
Clear cell cancer	16.2	30.2	0	53.5
Endometrioid cancer	22.4	14.4	1.6	61.6
Mucinous cancer	26.7	16.7	0	56.7

TILs, tumor-infiltrating lymphocytes; PD-L1, programmed death ligand 1.

## Possible Therapeutic Approaches in EOC That Could Transform a Cold Into a Hot Tumor

Currently, six PD-1/PD-L1 blockades have been approved by the FDA, including antibodies targeting PD-1 (nivolumab, pembrolizumab, and cemiplimab) or PD-L1 (atezolizumab, avelumab, and durvalumab) (**Table 2**). Recent clinical trials observed satisfactory survival results of PD-1/PD-L1 inhibitors in melanoma, non-small-cell lung cancer (NSCLC), head and neck cancer, and cutaneous squamous cell carcinoma (39–42). In contrast, in many recurrent solid tumors, the traditional treatment is dismal. For example, metastatic castration-resistant prostate cancer (mCRPC) is resistant to androgen deprivation therapy and is related to poor prognosis and limited therapeutic options (43). Recurrent EOC is the same case. However, in these patients, PD-1/PD-L1 inhibitors still do not seem to provide ideal results (44–46). As a single treatment, immune checkpoint inhibition (ICI) has a low response rate of only 10–35% (47).

**Table 2:** Six PD-1/PD-L1 inhibitors approved by the Food and Drug Administration (FDA).

Drug	Inhibited immune checkpoint
Atezolizumab	PD-L1
Avelumab	PD-L1
Durvalumab	PD-L1
Cemiplimab	PD-1
Nivolumab	PD-1
Pembrolizumab	PD-1

PD-1, programmed death-1 receptor; PD-L1, programmed death ligand 1.

Like most solid tumors at the advanced stage, recurrent or refractory EOC is always a cold tumor with a mild response to ICI immunotherapies and remains the most fatal gynecological cancer (48). **Table 3** summarizes the clinical studies investigating PD-1/PD-L1 inhibitors in recurrent EOC (49–63). In trials testing immune CPIs as monotherapy, the observed recurrent EOC response rate was 8.0-22.2%. As far as concerned, the KEYNOTE-100 study of phase II was the largest study of a single-agent immune checkpoint for recurrent EOC (51). In this study, 376 patients with recurrent EOC were enrolled. Pembrolizumab (200 mg) was given intravenously every 3 weeks. The overall response rate (ORR) was 8.0%, and the median progression-free survival (PFS) was 2.1 months.

**Table 3:** Finished clinical trials investigating the effects of PD-1/PD-L1 inhibitors in recurrent or refractory ovarian cancer.

PD-1/PD-L1 regimens	Trial number	Phase	Treatment	Numbers of OC patients	Enrolled patients	Survival results	Reference
Single regimen	NCT00729664	1	BMS-936559	17	Recurrent EOC	PR:6% (1/17; duration 1.3+ months.); SD:18% (3/17; duration 24+ weeks.)	(49)
	UMIN000005714	2	Nivolumab	20	Platinum-resistant EOC	CR: 10% (2/20, duration 11+ months); PR: 5% (1/20, duration 11+ months); SD: 30% (6/20, duration 11+ months)	(50)
	NCT02674061/KEYNOTE-100	2	Pembrolizumab	376	Recurrent EOC	ORR: 8.0%; Median PFS: 2.1 months	(51)
	NCT02054806/KEYNOTE-028	1b	Pembrolizumab	26	PD-L1–positive advanced metastatic ovarian cancer	ORR: 11.5%; Median PFS: 1.9 months; Median OS: 13.8 months	(52)
	NCT01772004/JAVELIN Solid Tumor Trial	1b	Avelumab	125	Recurrent or refractory EOC	ORR: 9.6%; Median PFS: 2.6 months; Median OS: 11.2 months	(53)
	NCT01375842	1	Atezolizumab	12	Recurrent EOC	ORR: 22.2%; Median PFS: 2.9 months; Median OS:11.3 months	(54)
Combined with CTLA-4	NCT02498600/NRG GY003	2	Arm 1: Nivolumab; Arm 2: Nivolumab + Ipilimumab	100	Persistent or recurrent EOC; PFI< 6 months	ORR: 12.2% vs 31.4%; Median PFS: 2 vs 3.9 months; Median OS: 21.8 vs. 28.8 months	(55)
Combined with chemotherapy	NCT02580058/JAVELIN Ovarian 200	3	Arm 1: Avelumab; Arm 2: PLD; Arm 3: Avelumab + PLD	566	Platinum-resistant/refractory EOC	ORR: 4% vs. 4% vs. 13%; DCR: 33% VS. 49% VS. 57%; Median PFS: 1.9 vs. 3.5 vs. 3.7 months; Median OS: 11.8vs. 13.1 vs. 15.7 months	(56)
	NCT02865811	2	Pembrolizumab and pegylated liposomal doxorubicin	26	Platinum- resistant/refractory EOC	CBR: 52.2%; ORR: 26.1%	(57)
	NCT02440425	2	Weekly paclitaxel + pembrolizumab	37	Recurrent EOC platinum- resistant EOC	ORR: 51.4%; DCR: 86.50%; Median PFS: 7.6 months; Median OS:13.4 months	(58)
Combined with PARPi	NCT02657889/TOPACIO/KEYNOTE-162	1/2	Pembrolizumab + Niraparib	60	Recurrent EOC	ORR: 18%; DCR: 65%; Median PFS: 3.4 months	(59)
	NCT02484404	2	Arm 1: Durvalumab + Olaparib; Arm 2: Durvalumab + Cediranib	19	Recurrent or metastatic EOC	ORR: 17% vs. 50%	(60)
	NCT02734004/MEDIOLA	1/2	Olaparib× 4w, then Olaparib + Durvalumab	34	gBRCAm platinum-sensitive relapsed	ORR: 63%; DCR: 81%	(61)
Combined with VEGFi ± chemotherapy	NCT02853318	2	Pembrolizumab + Bevacizumab + Oral metronomic cyclophosphamide	40	Recurrent platinum-sensitive/resistant/refractory	ORR: 47.5%; Median PFS:10 months	(62)
	NCT02873962	2	Bevacizumab + Nivolumab	38	Recurrent platinum-sensitive/resistant	ORR: 28.9%; DCR: 34.2%; Median PFS: 8.1 months	(63)

PR, partial response; SD, stable disease; CR, complete response; ORR, objective response rate; PFS, progression-free survival; OS, overall survival; CBR, clinical benefit rate; DCR, duration of response.

**Figure 4:**
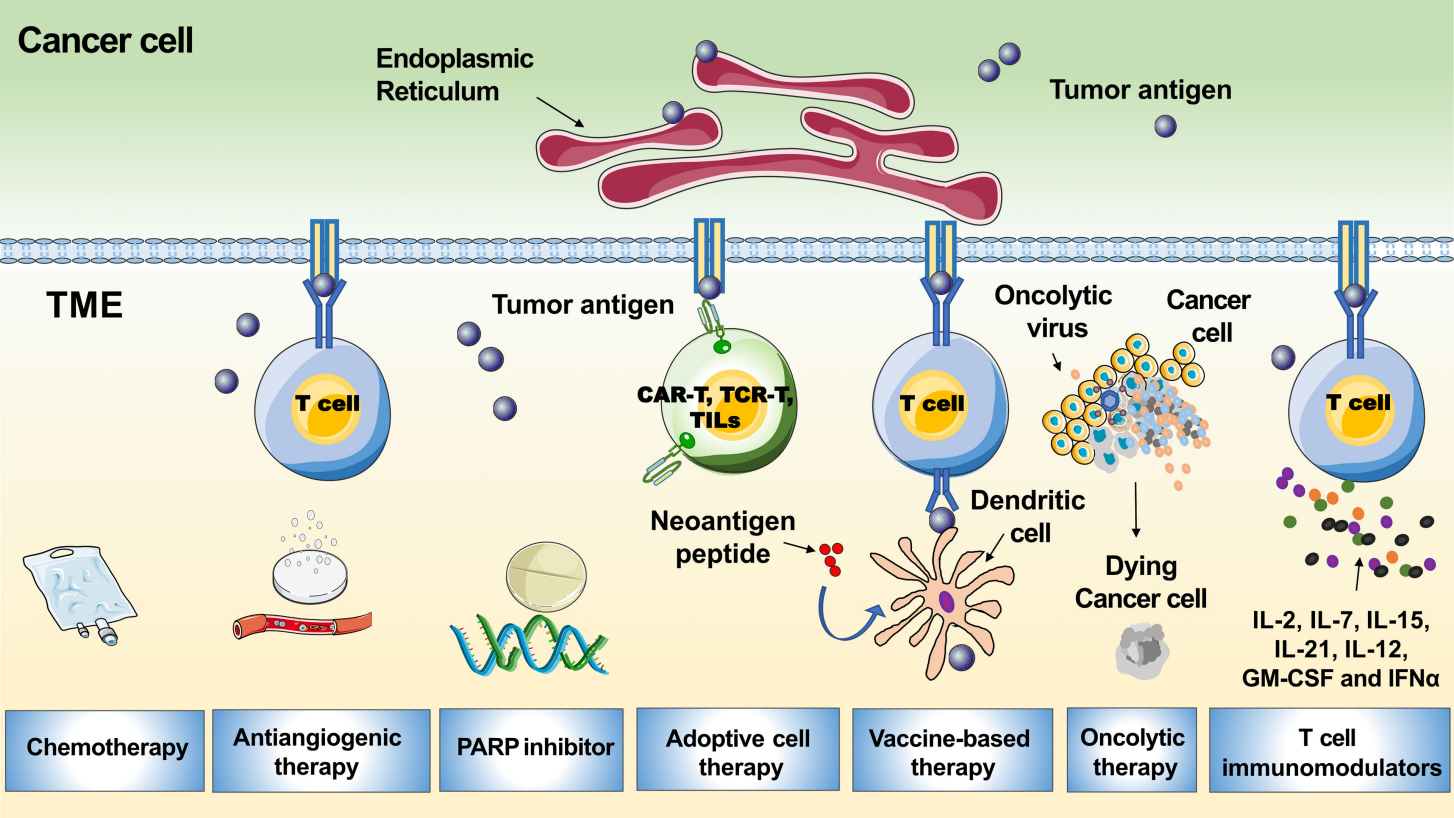
Tumor microenvironment related immunotherapeutic strategies in ovarian cancer. The graph shows multiple therapies combined with PD-1/PD-L1 blockades to boost the immune response, including chemotherapy, antiangiogenic therapy, PARP inhibitor, adoptive ​cell​ therapy, vaccine-​based ​therapy, oncolytic therapy and T cell ​immunomodulators.

To improve response rates, different combinations with anti-PD1-/PD-L1, including chemotherapy to release tumor antigens, antiangiogenic drugs to accelerate T-cell movement into the tumor, and improved lymph node effector T-cell priming and activation by anti-CTLA-4, DNA damage agents such as poly (adenosine diphosphate-ribose) polymerase inhibitors (PARPis) or vaccines to enhance T-cell reactivity against neoantigens, have been used (**Figure 4**) (64). Here, we summarize the latest developments in ICI combinations for recurrent EOC treatment. Furthermore, novel strategies that potentially strengthen immunotherapies for ovarian tumors are discussed.

### Chemotherapy

Studies in recent decades have demonstrated that chemotherapy has distinct effects on antitumor immunity (65). Immunogenic cell death might be promoted, and the immunogenicity of tumor cells could be increased under the effects of chemotherapy on tumor immunity. However, as the amount of neoantigen is very low, such an impact on the immunogenicity brought by chemotherapy might be insignificant (66).

Immunogenic dead tumor cells generated from chemotherapy release TSA and/or TAA along with damage-associated molecular patterns (DAMPs), which actively stimulate innate immune receptors and recruit immune cells into the TME (67). For example, cancer cell lines treated with anthracyclines could expose calreticulin on their surface during their immunogenic cell death. Such changes help DCs engulf cancer cells and promote tumor antigen presentation and tumor-specific CTL responses. Similarly, a standard dose of gemcitabine could increase the levels of class I or class II MHC molecules, thus augmenting the immune system response (68). Taxane and cyclophosphamide deplete MDSCs and Treg cells, respectively, reducing the inhibition of T-cell infiltration into tumors (69, 70). Dying tumor cells can also stimulate a Toll-like receptor (TLR) 3 dependent, cancer cell–autonomous type I interferon reaction and subsequently produce C-X-C chemokine ligand (CXCL) 10, leading memory T cells to the tumor bed (71, 72).

Meanwhile, chemotherapeutic drugs can induce a general immune response *via* off-target effects (73). 5-Fluorouracil could deplete tumor MDSCs, while cyclophosphamide could reduce Treg cells and promote the translocation of immune-stimulatory bacteria (74–76). Joalland et al. reported that the adoptive transfer of immune effector cells, consisting of allogeneic human Vγ9Vδ2T lymphocytes, is an effective goal for tracing and determining the spread of tumors in organisms. The results showed that chemotherapy and immunotherapy could be used together to improve the antitumor impacts (77). The impact between chemotherapy and immunotherapy is mutual. In fact, the immune status inside tumors also affects the efficacy of chemotherapy. Hot tumors are most likely more susceptible to chemotherapy than cold tumors (78). For example, tumor-associated macrophages (TAMs) are closely related to the progression and chemoresistance of EOC. The main tumor-promoting function of M2-like TAMs directly induces the invasion ability and chemoresistance of EOC cells by triggering its prosurvival signaling pathway (79). Paclitaxel and carboplatin have been the standard chemotherapy regimens for EOC for decades, both of which can induce apoptosis in tumor cells.

In summary, chemotherapy has an inhibitory effect on malignant tumors and participates in human immune system adjustment. Obvious survival benefits from the combination of platinum chemotherapy with pembrolizumab have been observed in a recent trial of lung cancer (80). The success of anti-PD-1 therapy combined with chemotherapy in the treatment of metastatic NSCLC demonstrated the advantages of this dual approach. It is hopeful that chemotherapy could improve the immunotherapy effects in EOC *via* tumor immune microenvironment modification. A preclinical model of EOC confirmed that the number of activated T cells and DCs and the expression of cytotoxic factors can be increased by combining chemotherapy (paclitaxel + carboplatin) and immunotherapy (anti-IL-10, 2’ 3’-cGAMP + anti-PD-L1) (81). To evaluate the impact of ICI on follow-up therapy, Liu conducted a retrospective study that recruited 79 patients with recurrent EOC. This result suggested that ICI could improve the subsequent chemotherapy outcome. The most commonly used cytotoxic drugs included taxane, platinum, and pegylated liposomal doxorubicin. The median OS of 18.3 (95% CI, 11.8–22.7) months from initiation of first treatment after ICI is promising compared to the inferior outcomes of other recent studies (82). In another clinical trial, Wehnham et al. evaluated 37 patients with platinum-resistant EOC treated with weekly paclitaxel (80 mg/m^2^) and pembrolizumab (200 mg q3 w); the ORR was 51%, and the 6-month PFS was 52% (58). Lee’s phase II trial showed that the ORR and median PFS of the combination of pembrolizumab with pegylated liposomal doxorubicin in platinum-resistant EOC could be improved compared to PLD or anti-PD-1/PD-L1 agents alone in the past (57). The study observed 12 patients who achieved clinical benefit with a clinical benefit response (CBR) of 52.2% (95% confidence interval (CI), 30.6-73.2%). There were five partial responses (PRs) (21.7%) and one complete remission (CR) (4.3%), and the ORR was 26.1%. Six patients had stable disease (SD) lasting at least twenty-four weeks. However, there was no significant correlation between PD-L1 staining and objective response, PD-L1 staining and TIL score, or TIL score and objective response. To date, the largest trial with reported data in platinum-resistant ovarian cancer is the JAVELIN Ovarian 200 trial, a three-arm randomized phase III trial that enrolled 566 patients. Their results showed that neither avelumab plus pegylated liposomal doxorubicin (PLD) nor avelumab alone significantly improved progression-free survival or OS versus PLD. However, retrospective subgroup analysis including 442 tumor samples revealed that patients with tumors expressing PD-L1 may benefit from avelumab + PLD. PD-L1+ patients who received avelumab combination therapy had a trend toward prolonged PFS and OS (PFS: 3.7 vs. 3 months, P = 0.0143; OS: 15.7 vs. 13.1 months, P = 0.2082) (56).

These studies suggest that the synergy of cytotoxic therapy and PD-1/PD-L1 blockade may be beneficial and feasible in the treatment of EOC in selected patients. Biomarker analysis including PD-L1 and CD8 expression might predict benefit with chemotherapy and immunotherapy treatment in ovarian cancer.

### Antiangiogenic Therapies

Vascular abnormality is one distinguishing feature of many solid tumors, including EOC. Angiogenesis and immunosuppression have a close connection (83). Angiogenesis facilitates immune escape, which is involved in nonpathological tissue repair as a physiological mechanism promoting cancer development (84). Many proangiogenic molecules can suppress the immune system, such as antigen presentation, T-cell trafficking, T-cell priming and T-cell tumor infiltration (85). Thus, antiangiogenic drugs would be helpful in improving the antitumor immune response. The interactions of anti-angiogenesis and immune systems include 1) direct effects when binding to their cognate receptors expressed by immune cells; 2) indirect effects when they induce changes in endothelial cell protein expression; and 3) indirect physical effects by promoting vascular normalization or reducing angiogenesis (86).

In advanced-stage melanoma, vascular endothelial growth factor (VEGF) is elevated, which is associated with negative immune effects, such as impaired dendritic cell function. It was also linked with both elevated and decreased T helper 2 (Th2) cytokines. These effects were found to result in suppression of effective antitumour immunity (87). Patients with higher levels of serum VEGF were found to have worse OS with ipilimumab treatment, which provided a rationale for targeting VEGF (88). Compared with vaccination alone, antiangiogenic therapy combined with vaccination or adoptive cell transfer increased T cells in the tumor, thereby enhancing anticancer activity (89). Moreover, the normalization of blood vessels was related to the increase in CD4+ T helper type 1-cell (Th1) cell accumulation and anticancer activity (90). Nevertheless, VEGF can deplete T cells by upregulating the expression of PD-1 on T cells (91). Cytotoxic effects were observed in the colorectal cancer model CT26 mice treated with PD-1 blockade and anti-VEGF drugs. The tumor growth reduction rate of the experimental group was as high as 75% compared with that of the control group. In the same way, in mice with tumors derived from colon cancer C26 cell injection, the combined use of anti-VEGFR2 and anti-PD-1 MABs enhanced the effects of tumor growth inhibition (92). In EOC, Zhang et al. found that PD-L1 and two angiogenesis-related proteins (VEGF and Semaphorin4D) were overexpressed in cisplatin-resistant EOC patients. *In vitro* studies demonstrated the synergistic effects of atezolizumab and bevacizumab in inhibiting the proliferation and metastasis of cisplatin-resistant EOC cell lines (93). This result provides an additional methodology for the treatment of cisplatin-resistant recurrent EOC.

Liu et al. conducted a phase 2 study to evaluate the activity of combined nivolumab and bevacizumab in women with relapsed ovarian cancer. The ORR was 40.0% (19.1%-64.0%) in platinum-sensitive participants and 16.7% (95% CI 3.6%-41.4%) in platinum-resistant participants. Thus, it is possible that bevacizumab activity is higher in platinum-sensitive patients. Interestingly, better response rates were observed in patients with PD-L1–negative tumors in this study, suggesting that PD-L1 expression unreliable in predicting the response to immunotherapy activity (59). Similarly, Zsiros et al. reported one open-label, single-arm phase 2 cohort study with 40 recurrent EOC patients enrolled. Thirty patients (75.0%) had platinum-resistant disease, and 10 (25.0%) had platinum-sensitive disease (63). The regimen was intravenous pembrolizumab 200 mg, bevacizumab 15 mg/kg every 3 weeks, cyclophosphamide 50 mg orally once daily at the same time. The ORRs in platinum-resistant and platinum-sensitive patients were 43.3% and 60.0%, respectively, with a total ORR of 47.5%. When patients were analyzed based on the number of prior chemotherapy lines, patients with no more than 3 prior chemotherapy lines had a significantly longer PFS. This result revealed that earlier introduction of immunotherapy in the EOC may lead to greater PFS benefit. Moreover, several ongoing trials are carried out to evaluate the combined effects of antiangiogenic and anti-PD-1/PD-L1 agents. In the recent GOG 3015/ENGOT OV-39/IMagyn050 trial, patients with FIGO stage III and macroscopic residual disease who underwent primary major surgery or those with stage IV disease were enrolled in the trial. The participants received chemotherapeutic strategies of paclitaxel, carboplatin and bevacizumab, then with maintenance of bevacizumab. Subsequently, they were randomized to receive an atezolizumab 1200 mg dose every 3 weeks or placebo during the chemotherapy and maintenance phases. A total of 1301 patients were recruited. In patients receiving atezolizumab vs. placebo, the median PFS was 19.5 vs. 18.4 months (P =0.28) in the intention-to-treat population and 20.8 vs. 18.5 months (P =0.038) in the PD-L1-positive population. In the midterm (immature) OS results, atezolizumab had no obvious effect. However, the effects of combined immunotherapy and bevacizumab in EOC still need to be further explored (94).

### PARPi

PARPis are one of the most representative new therapies for EOC, targeting the DNA repair fragility of tumor cells. In homologous recombination deficiency (HRD) tumors, double-strand breaks cannot be accurately repaired in the presence of PARPis (95). Tumor cells are destroyed in the accumulation of unrepaired DNA damage. Among the PARP inhibitors, olaparib, niraparib, and rucaparib trap PARP approximately 100-fold more efficiently than veliparib, while talazoparib appears to be the most potent PARP trapper investigated thus far. Increased PARP trapping is associated with high myelosuppression, which results in variation of the recommended doses across PARP inhibitors (96). Since PARPis could increase the burden of neoantigens and activate DNA damage-associated innate immune pathways in tumor cells, the strategies of combining PARPis and checkpoint inhibition will probably be feasible. This idea is supported by Jiao’s study (97). They found that PD-L1 overexpression in breast cancer cell lines and animal models after PARPi treatment, as PARPi, inactivated GSK3 β. The overexpression of PD-L1 reduced the effects of PARPi. The application of PD-L1 blockade again improved the T-cell killing effects of PARPi. This combined therapy significantly improved the therapeutic effect *in vivo* compared to using each drug separately. Appleton’s research examined the effectiveness of using three-dimensional (3D) ellipsoids to detect patient-specific immune-related activities in EOC (98). They found that pembrolizumab increased the secretion of cytokines in a T-cell-dependent way, enhanced the cytotoxicity of olaparib, and reduced the viability of the spheroids. The combination of durvalumab and olaparib could kill cancer cells through a synergistic effect. This work demonstrates the effectiveness of ICIs in combination with PARPis in a preclinical setting.

Several phase II trials have already yielded encouraging results of PARPi and immunotherapy combinations in recurrent EOC patients. The MEDIOLA trial demonstrated promising antitumour activity and safety of the combination of olaparib and durvalumab compared to monotherapy studies (62). The study was carried out on 34 germline breast cancer susceptibility gene (BRCA)-mutated platinum-sensitive recurrent EOC patients. They found that the ORR and 12-week DCR of the combination of olaparib and durvalumab in these patients were 72% and 81%, respectively. The median PFS was 8.2 months (95% CI 4.6–11.8), and the median OS was 21.5 months (95% CI 16.2–25.7). TOPACIO/KEYNOTE-162 was a single-arm phase 1/2 clinical trial that enrolled patients regardless of BRCA gene status. Patients with advanced triple-negative breast cancer (TNBC) or recurrent EOC received niraparib and pembrolizumab. The ORR of the EOC cohort (n = 62 patients) was 18% (90% CI, 11–29%), and the disease control rate (DCR) was 65% (90% CI, 54–75%) (60). Note that PD-L1 expression was not related to the treatment effects. Participants with only one prior cytotoxic therapy had a better response rate. The most common treatment-related adverse events (AEs) were fatigue, nausea, iron deficiency anemia and severe constipation. In addition, Färkkilä et al. found that mutation feature 3 reflected defective homologous recombination DNA repair and had a better immune score (99). The results of immunogenomic analysis of tumor samples from the TOPACIO study indicated that a positive immune score could predict the efficacy of the niraparib/pembrolizumab combination. Exhausted CD8+ T cells could be predictive of the response to the niraparib/pembrolizumab combination (100). The clinical data strongly support the strategy of dual targeting PARP and PD-1.

### Adoptive Cell Therapy

The elimination of tumor cells depends largely on T cells. In recent years, T-cell adoptive immunotherapy has been developing rapidly. Adoptive T-cell therapy includes the autologous or allogeneic transplantation of TILs or genetically modified T cells with chimeric antigen receptors (CARs) or tumor-specific T-cell receptors (101). This personalized strategy is particularly promising for cancer patients who do not respond well to conventional therapies.

Adoptive cell therapy with TILs has been proven effective in many cancers, especially malignant melanoma, with ORRs ranging from 40–70% (102–104). TIL therapy demonstrates the effects of eliminating solid tumors with high mutational burden malignancies such as melanoma. Studies of melanoma have found that TILs are now considered an abundant source of effector T cells, which exhibit tumor recognition. The TILs isolated from the patient’s tumor tissue are reinfused after *in vitro* activation and expansion, which represents the ultimate personalized therapy. In recent decades, TIL-based adoptive cell therapy in EOC has been extensively studied. Many studies suggest that the presence of the preferential intraepithelial location of TILs is a good predictor for EOC. Aoki et al. published the first trial of TIL therapy using TILs expanded in IL-2 for advanced or recurrent EOC cases in 1991 (105). Five out of 7 patients showed clinical benefits after receiving TILs without chemotherapy. Meanwhile, 9 out of 10 patients showed clinical benefits after receiving chemotherapy before TIL infusion. This study demonstrated the effects of TIL therapy in EOC for the first time. Another study in Japan evaluated the clinical benefits of TIL infusion with combination chemotherapy for EOC patients after primary debulking surgery. Patients receiving TILs had better OS than those whose TILs did not expand to sufficient numbers. Their results suggested that the adoptive transfer of TILs probably induced immunoactivation of cellular immunity and enhanced natural killer activity in EOC (106). One pilot study enrolled six patients with progressive platinum-resistant metastatic EOC. The participants received chemotherapy, followed by TIL infusion and intravenous IL-2. The treatment was well tolerated, and the toxicities were durable. Four patients had SD for three months, and two patients had SD for five months, with five patients with the target lesion size decreasing. Antitumor reactivity was observed in TIL infusion products from five patients, but no antitumor reactivity was detectable in peripheral blood lymphocytes collected after treatment (107). In addition, some experiments showed the fusion of CPI and adoptive T-cell transfer (ACT) in EOC patients. In one trial, six patients with advanced metastatic high-grade serous EOC were treated with immunotherapy including ipilimumab followed by surgery to obtain TILs and infusion of REP-TILs (TILs expanded in the rapid expansion protocol), low-dose IL-2 and nivolumab. One patient had PR, and the other five had SD for 12 months (108).

CAR-T-cell therapy is another representative of adoptive T-cell immunotherapy. It has the advantages of high specificity, not being limited by MHC, and long-term efficacy after infusion. CAR-T cells are a combination of single-chain variable fragments (scFvs) created by gene transduction that recognize tumor-associated antigens and activation motifs of T cells (109). CAR-T cells have been shown to have a good therapeutic effect on hematologic cancer (110). Recently, Xu et al. described a method that involved a bispecific CAR-T targeting two different B-cell maturation antigen (BCMA) epitopes, combining two camel-derived antigen-binding domains in the construction of a single CAR in patients with multiple myeloma. They achieved the highest CR rate of 76% (13/17) (111). Compared to blood tumors, solid tumors respond poorly to CAR-T-cell therapy. However, several studies with small samples have confirmed the effects of CAR-T cells in EOC. The surface antigens targeted by CARs are mainly proteins and glycolipids (112). In CAR-T-cell therapy for recurrent EOC, the most common target antigens consist of Mucin-16 (MUC-16), mesothelin, human epidermal growth factor receptor-2 (HER-2) and folate receptor-alpha (FR-α). MUC-16 is overexpressed in more than 80% of EOCs (113). *In vitro* experiments showed that MUC-16-CAR-T cells have specific killing effects on MUC-16^+^ EOC cells. Intravenous or intraperitoneal administration of MUC-16-CAR-T cells can inhibit the growth and development of cancer cells and mouse tumor-bearing solid models (114). Mesothelin is a glycoprotein anchored to the plasma membrane according to the phosphatidylinositol region (GPI). It also rises in the pleura, peritoneum, pericardium, mesothelium and EOC cells (115). Beatty et al. reported two malignant pleural mesothelioma patients who received adoptive transfer of mRNA CAR-T cells that target mesothelin (CARTmeso cells) (116). After intravenous injection, CAR T cells existed temporarily in the blood and moved to cancer regions. The treatment demonstrated clinical and laboratory benefits of antitumor activity and no overt off-tumor on-target toxicity. Oberg developed the bispecific antibody [(HER-2)2xCD16] in the tribody format, which could redirect CD16-expressing γδ T lymphocytes and NK cells to the tumor-associated cell surface antigen HER-2 to improve their cytotoxic antitumor activity. In HER2-expressing cancer cells, compared with trastuzumab, tribody[(HER-2) 2xcd16] was better at inducing γδ T-cell- and NK-cell-mediated lysis, including pancreatic ductal adenocarcinoma (PDAC), breast cancer and EOC (117). However, most CAR-T-cell treatments in EOC are still in the experimental stage. The central issue hindering development was that CAR-T cells could not persist and expand *in vivo* for more than a few days (118). Efforts to overcome this problem by increasing the dose have failed and have greatly restricted the application of CAR-T cells in clinical practice (119). To obtain a durable clinical response to cell-based gene therapy, permanent transgene expression is indispensable. Murine gamma retroviruses and lentiviruses are two vector systems applied in clinical gene therapy that may provide long-term CAR transgene expression (120). Recent data showed that the expression levels of T-cell coinhibitory receptors, such as PD-1 and Tim-3, were upregulated on CAR-T cells (121). The PD-L1/PD-1 pathway was able to directly inactivate CD28 signaling in CAR-T cells and therefore inhibit CAR-T-cell function (122). Taken together, PD-1/PD-L1 inhibitors and CAR-T therapy might exert synergistic antitumor effects.

Compared to CARs binding unprocessed tumor surface antigens without MHC processing, TCR therapy addresses both tumor intracellular and surface antigenic peptides embedded in MHC. TCRs target special antigens to boost the efficacy of anticancer immunotherapy (123). Many TAAs could be used as targets for immunotherapy. These include the cancer testis antigens (e.g., New York Esophageal Squamous Cell Car- cinoma-1 (NY-ESO-1)), differentiation antigens (e.g., Melanoma antigen family A-3 (MAGE-A3)), overexpressed oncogenes (e.g., Wilms’ Tumor antigen 1 (WT1)), tumor suppressor genes (e.g., Tumor Protein P53(TP53)); and TAAs that are organ-specific antigens that are transiently expressed. Moreover, oncogenic viruses, such as HPV, EBV, and HBV, are promisingly targeting antigens, which could induce eradication to virus-induced cancer cells. TCRs have several structural advantages, such as more subunits in their receptor structure, greater immunoreceptor tyrosine-based activation motifs (ITAMs), less dependence on antigens and more costimulatory receptors (such as CD3, CD4, and CD28) (124). NY-ESO-1 is a cancer testis antigen and a promising target for immunotherapy that is overexpressed in approximately 40% of non–small cell lung, ovarian, and melanoma cancers and in greater than 70% of certain sarcoma subtypes (125, 126). It has been found to be able to trigger spontaneous humoral and cellular immune responses. NY-ESO-1-directed vaccination has obtained some promising clinical results in early phase I/II studies, and adoptive cell therapy has resulted in partial responses (PRs) in 60% of NY-ESO-1-expressing sarcomas in small phase I trials (127). Somaiah et al. conducted a first-in-human, open-label phase I trial to examine the clinical safety and preliminary efficacy of LV305 in patients with advanced sarcoma or other solid tumors expressing NY-ESO-1 (128). Overexpression of NY-ESO-1 in EOC relates to poor clinical outcomes (129). A phase I, open-label study assessed the safety and *in vivo* immunogenicity of synthetic overlapping long peptides (OLPs) from NY-ESO-1 in 28 relapsed EOCs. They found that OLP could rapidly induce consistent integrated immune responses with CD8+ and CD4+ antibodies. However, the induction of NY-ESO-1 antibody and NY- ESO-1 CD8+ and CD4+ T-cell responses was similar in patients with and without NY-ESO-1 tumor expression. Somaiah et al. conducted a first-in-human, open-label phase I trial to examine the clinical safety and preliminary efficacy of LV305 in patients with advanced sarcoma or other solid tumors expressing NY-ESO-1 (128). LV305 is a novel lentiviral-based cancer vaccine that induces the expression of NY-ESO-1 cancer testis antigen in dendritic cells. Patients were enrolled with sarcoma (n= 24), ovarian (n= 8), melanoma (n = 6), and lung cancer (n=1). All the participants received LV305 (1 mL per dose) intradermally once every 3 weeks. The disease control rate was 56.4% in all patients and 62.5% in sarcoma patients. A *post hoc* exploratory analysis revealed significantly longer OS in patients with increased T-cell clonality in posttreatment peripheral blood mononuclear cells among all patients (P = 0.0116)), indicating that LV305-induced clonal expansion of T-cell anti–NY-ESO-1 responses may be associated with OS. Further development of LV305 is planned in combination with G305 as the combination therapy “CMB305.” G305 is a cancer vaccine composed of a full-length recombinant NY-ESO-1 protein and the synthetic TLR4 agonist glucopyranosyl lipid An in a stable emulsion (130). Studies of CMB305 alone or in combination with the anti–PD-L1 checkpoint inhibitor atezolizumab are ongoing (NCT02609984, NCT02387125).

Although clinical data on the combination of ICB and ACT are currently limited in EOC, the field of immune therapy has the potential to reach better cancer care.

### Vaccine-Based Therapy

Ovarian cancer cells affect the immune system by coordinating the molecular signal network exposed or secreted on the surface, causing T-cell exhaustion and DC dysfunction (131). DC dysfunction may still prevent the complete recovery of immune function, although treatment with immune CPIs can reverse tumor-induced T-cell exhaustion. This problem can be solved by inoculation with functional antigen-presenting cells (APCs).

DCs carry out antigen-specific immune responses, phagocytosis, processing and presentation of foreign and pathogen-related antigens on their cell surface (132). Therefore, DCs hold great potential for becoming effective cancer vaccines in the adjuvant setting. Tumor antigens include tumor cell lysate and tumor-associated peptides/proteins. DCs present tumor-associated antigens to activate T cells by MHCI/II molecules (133). DC vaccines are regarded as a promising strategy. A phase III trial is currently determining the effectiveness of an RNA-loaded autologous DC vaccine as an adjuvant vaccination in treating uveal melanoma (NCT01983748). Although the DC vaccine is generally safe and has extremely low toxicity, it has shown limited clinical benefits thus far and rarely achieves an objective clinical response of no more than 15% in most indications (134). One explanation for the low response rate is that patients with large tumor burdens have an immunosuppressive TME and have immune tolerance to their tumors. Therefore, the number of clinical trials of the combination of DC vaccines with other drugs has increased dramatically.

In one trial, autologous DCs pulsed with HOCl-oxidized autologous tumor lysate (OCDC vaccine) were obtained from patients as a personalized vaccine. Patients received 5 dose injections of DC vaccine every two weeks (~5-10*10^6^ DCs/dose), followed by maintenance treatment every month until disease progression or vaccine supply exhaustion. Five patients with recurrent EOC (aged 48-63 years) were included in the clinical study. Two subjects (S2 and S3) had no evidence of disease (NED) after recurrence. Three participants (S1, S4 and S5) were admitted to the trial with radiographically measurable tumors. Two of them progressed, and the other one showed a mixed response according to the solid tumor efficacy evaluation specification (RECIST). The durable PFS times were 36 months and 44 months. All the data showed the efficacy of DC vaccine treatment in EOC patients (135).

Rodney Rocconi and colleagues reported another vaccine strategy (136). Whole tumor lysates prepared at primary or interval debulking surgery were used to create tumor-specific EOC vaccines. After randomization, patients received maintenance vaccination (gemogenovatucel-T) or placebo after complete clinical remission. The subgroup analysis showed that BRCA wild-type EOC patients had an outstandingly longer recurrence-free survival than the control group (HR 0.51, 90% CI 0.30–0.88; P=0.020). The 1-year and 2-year OS of vaccinated BRCA wild-type disease patients were significantly longer than those of unvaccinated patients. Autologous tumor vaccines could be a new and personalized maintenance strategy for patients with EOC. This strategy is especially necessary for the overwhelming majority of BRCA wild-type EOC patients. However, the recurrence-free survival rate was not significantly improved (11.5 months in the vaccine group vs. 8.4 months in the placebo group; P=0.078). Nonetheless, one multicenter clinical trial VITALIA will begin (NCT 03905902). In the study, during the induction period, EOC patients will receive a DC vaccine named DCVAC/OVCA or placebo combined with platinum-based chemotherapy, regardless of whether bevacizumab was used. In the maintenance period, patients will be injected with the DC vaccine combined with bevacizumab and PARPi or only supportive treatment (137).

Kadam et al. applied an anti-PD-1 plus CCL21-DC tumor lysate vaccine in a murine lung cancer model. The expression of perforin and granzyme B in the TME, as well as TIL activity, were increased. Mice with treatment-induced tumor eradication developed immunological memory, leading to cancer recurrence-free survival (138). Since PD-1/PD-L1 blockade will increase the amplitude of therapeutic cancer vaccine-mediated activated T-cell responses in the TME, therapeutic cancer vaccines combined with PD-1/PD-L1 immune checkpoint blockade therapy could be a reasonable approach with the potential for cancer-free survival (139).

### Oncolytic Therapy

An oncolytic virus (OV) is a virus that exists naturally or can be obtained by recombination. The virus eliminates cancer cells and targets tumor blood vessels through oncolytic action, direct action or immune-mediated action to selectively kill cancer cells and related stromal cells. Clinical trials of viruses in cancer treatment are needed to advance this field (140). In addition to direct and nearby antitumor activity, OVs can induce more powerful, systemic and durable antitumor immunity (141). Tumor cells undergoing apoptosis cause an effective antitumor immune response, releasing TAAs and additional DAMPs. More importantly, the treatment exerts therapeutic effects not only at the local site but also at distant tumor sites. The OVs must retain their immune stimulation ability without toxic factors to be used for treatment. Many viruses have been developed and evaluated in clinical research, including adenovirus, poxvirus, measles virus, herpes simplex virus type1 (HSV-1), poliovirus, Coxsackie virus, Newcastle disease virus (NDV) and reovirus (142).

Havunen established oncolytic adenoviruses coding for human IL-2, tumor necrosis factor-α (TNF-α), or both. According to the results of the study, they found that these viruses showed strong antitumor effects on human tumors in severe combined immune deficiency (SCID) mice. The treated animals were protected from tumor recurrence, which indicated a memory response. They considered that cytokine-armed oncolytic adenoviruses could change the TME to synergize with adoptive cell therapy (143). Santos cultivated fresh tumor cultures *ex vivo* originating from patients with advanced EOC and confirmed that the oncolytic adenovirus Ad5/3-E2F-D24-hTNFa-IRES-hIL2 was adapted to enhance antitumor TIL responsiveness by reconnecting to the ovarian TME (144). Another OV, granulocyte macrophage colony stimulating factor (GM-CSF)-coding oncolytic adenovirus, was given to patients with solid tumors refractory to standard therapies in one clinical trial. Among the 37 EOC patients, treatment was generally well tolerated. Intestinal problems were the most common adverse events. No grade 4 or grade 5 adverse events were found in 12 patients. Only grade 3 adverse events were reported. The whole cancer control rates were 56% and 55% with imaging (RECIST1.1 or PET criteria) and CA125 detection, respectively (145).

Although the expression of PD-L1 is very low in many solid tumors, recent data have shown that oncogenic viruses can induce chronic inflammation and secrete cytokines such as IFN-γ, which upregulate PD-L1 expression in the TME as a means of self-protection (146–148). Therefore, expanding the application of OV and PD-1/PD-L1 inhibitors would be inspiring. Liu et al. developed a vaccinia virus expressing CXCL1, which could enhance T-cell infiltration into the tumor and upregulate the expression of PD-L1. Combined treatment of OV with anti-PD-L1 finally elicited systemic and potent antitumour immunity in tumor models in B6 mice. The combination therapy led to over 40% cure in aggressive models of peritoneal carcinomatosis from colon and ovarian cancers (142).

In summary, adenoviruses could generate new T-cell responses against tumor antigens and neoantigens in preclinical models and clinical trials. Recent preclinical data have shown that the combination of OV and PD-1/PD-L1 inhibitors could work synergistically to exert cytotoxicity to cancer cells, eliminate immunosuppressive cells and elicit more potent and sustained systemic antitumor immunity. Therefore, expanding the successful application of oncolytic therapy would be significant in recurrent EOC.

### T-Cell ​Immunomodulators

The therapeutic effects of cytokines such as IL-2, IL-7, IL-12, IL-15, IL-21, GM-CSF and interferon-α (IFN-α) have been evaluated in many clinical trials. They regulate the function of tumor-killing T cells in direct or indirect ways (149). One main reason that patients do not benefit from PD-L1 blockade is the

absence of preexisting tumor inflammation (150). Therefore, a strategy to sensitize the response to PD-L1 blockade could include pretreatment with an immunotherapy that induces intratumoral T-cell infiltration. Mice lacking IL2 die from lethal T-cell–driven autoimmunity due to reduced functional Tregs, indicating that IL2 signaling is essential for excessive T-cell activation (151).

IL-2 is a cytokine produced by activated CD4+T cells that activates the proliferation of CD8+T cells and CD4+ Tregs (152). Despite its contradictory effects, IL-2 treatment received FDA approval in 1998. Aldesleukin, recombinant IL-2, achieved an ORR of 16% and a CR rate of 6% in patients with advanced melanoma in the first trial (153). One phase I/II study evaluated the safety and feasibility of autologous DCs and IL-2 in EOC patients with minimal residual disease. The enrolled patients received two subcutaneous injections of DCs derived from autologous monocytes pulsed with autologous tumor lysate and keyhole limpet hemocyanin (KLH) every 4 weeks, 4 months after initial debulking and chemotherapy (154). Then, a low dose of IL-2 (200 mIU) as an immune adjuvant was given for 14 consecutive days. Among the 10 patients, 3 showed CR after DC vaccination for 83, 80.9 and 38.2 months and no disease recurrence. One patient with SD had a tumor that completely disappeared after DC vaccination and continued for 50.8 months without recurrence. Alternative IL-2 structures are under study to improve the effects and attenuate toxicities. NKTR-214 (bempegaldesleukin) is a drug of recombinant IL-2 coupled with 6 releasable polyethylene glycol (PEG) chains. By PEGylating IL-2, the binding ability of the IL2Ra receptor is reduced, and the half-life is longer. Its affinity for Tregs is also reduced (155). Several phase I/II and III trials tested NKTR-214 as a single treatment or combined immune checkpoint blockade in melanoma (NCT03635983, NCT03138889). The results of the phase I/II PIVOT-02 study of NKTR-214 combined with nivolumab (NCT02983045) showed that in 11 patients with advanced melanoma, the DCR was 91%, the ORR was 64%, and the toxicity was mild (156).

Drerup found that IL2 receptor β-selective IL2/anti-IL2 complexes (IL2c) could preferentially stimulate effector T cells in an orthotopic mouse ID8 aggressive ovarian cancer model (157). IL2c obviously lowered the tumor microenvironmental CD8+/Treg ratio and induced a fragile Treg phenotype. The combination of anti-PD-L1 antibody and IL2c could generate complete tumor regression and protective immune memory, which could not be achieved by either monotherapy. These data support the application of IL2c combined with other immunotherapies to enhance antitumor immunity.

IL-15 can effectively promote the activation and proliferation of CD8+T and NK cells. It did not cause capillary leak syndrome in the preclinical model and had acceptable toxicity in the IL-15 subcutaneous injection test (158). *In vivo*, IL-15 has a short half-life time and lacks potent transpresentation by IL-15 receptor α (IL-15Rα). N-803 is an IL-15 superagonist developed by ImmunityBio that combines IL-15 with an activating mutation, an IL-15Rα sushi domain for trans-presentation, and IgG1-Fc for increased half-life. Experiments showed that N-803 could increase IFNγ-induced CXCL10 secretion and target killing after increasing exposure time (159). Unfortunately, no significant benefits of IL-15 have been observed in clinical studies until now (160). However, some studies found that IL-7 and IL-15 might be better than IL-2 in adoptive cell therapy (161).

## Conclusions

Although changing a cold tumor into a hot tumor remains a great challenge, an increasing number of pioneer studies focusing on the mechanisms of the immunosuppressive microenvironment and PD-1/PD-L1 blockade combination treatment have shed light on a brilliant future of EOC treatment. Our review provides an overview of valuable evidence and significant therapeutic strategies for PD-1/PD-L1 blockade in recurrent EOC. Many studies have demonstrated that PD-1/PD-L1 blockade therapy is a safe treatment in patients with EOC, but monotherapy showed only modest efficacy in recurrent EOC. This may demonstrate the limited immunity and immunosuppressive microenvironment in EOC. Overall, recurrent EOC is a cold tumor. Its conversion into an inflamed tumor will require a prior combination of therapies to induce immune infiltration, followed by immune checkpoint modulators to remove the breaks. To enhance immunity in EOC, combining a priming therapy to increase T-cell responses, including DC-based vaccination, would be rational. Moreover, according to the preclinical and clinical data, immunotherapy combined with chemotherapeutics, antiangiogenic drugs or DNA demethylating agents can stimulate anticancer immunity by various mechanisms and hence may be beneficial in patients with cold EOC.

Another tremendous challenge impeding the application of immunotherapies in recurrent EOC is the lack of comprehensive knowledge of the cancer-immune interaction parameters. How to use standardized methods to measure the individual immunity parameters is lacking. Additionally, accurate algorithms of treatment depending on different tumor or TME characteristics should be developed to select adequate immunotherapy strategies. To solve these problems, the development of biomarkers to determine these therapies effectively is critical, and biomarker-guided clinical trials will be necessary to tailor these approaches to EOC patients. Tumor genomic profiling with immune profiling will help to provide a more comprehensive understanding of an individual patient’s tumor and select the accurate treatment.

## Author Contributions

SY and MC designed the manuscript. MC, YZ, and QC wrote the manuscript. MC, YZ, MX, and DL drew the figures and tables. SY mainly revised the manuscript. All authors contributed to the article and approved the submitted version.

## Funding

This study was funded by the National Natural Science Foundation of China 81602261; CSCO Cancer Research Foundation (Y-sy2018-120); Beijing Kanghua Foundation (KH-2021-LLZX-052). The funders had no role in the study design, data collection and analysis, decision to publish, or preparation of the manuscript.

## Conflict of Interest

The authors declare that the research was conducted in the absence of any commercial or financial relationships that could be construed as a potential conflict of interest.

## Publisher’s Note

All claims expressed in this article are solely those of the authors and do not necessarily represent those of their affiliated organizations, or those of the publisher, the editors and the reviewers. Any product that may be evaluated in this article, or claim that may be made by its manufacturer, is not guaranteed or endorsed by the publisher.

## Glossary

**Table:**

ACT	adoptive T-cell transfer
BCMA	B-cell maturation antigen
BRCA	Breast Cancer Susceptibility Gene
CAF	cancer-associated fibroblast
CAR	chimeric antigen receptor
CBR	clinical benefit response
CPI	checkpoint inhibitor
CSC	cancer stem cell
CTLA-4	cytotoxic T lymphocyte associated antigen-4
CTL	cytotoxic T lymphocyte
CXCL	C-X-C chemokine ligand
DAMP	damage-associated molecular pattern
DC	dendritic cell
DCR	disease control rate
EOC	epithelial ovarian cancer
FDA	Food and Drug Administration
FR-α	folate receptor-alpha
GM-CSF	granulocyte macrophage colony stimulating factor
GPI	phosphatidylinositol region
HER-2	human epidermal growth factor receptor-2
HLA	human leukocyte antigen
HSV-1	herpes simplex virus type 1
IFN-α	interferon-α
IL	interleukin
LAG3	Lymphocyte activation gene-3
MAB	monoclonal antibody
MAGE-A3	Melanoma antigen family A-3
MDSC	myeloid-derived suppressor cell
MUC-16	Mucin-16
NDV	newcastle disease virus
NED	no evidence of disease
NK	natural killer
NYESO-1	New York Esophageal Squamous Cell Carcinoma-1
ORR	overall response rate
OS	overall survival
OV	oncolytic virus
PARPi	poly (adenosine diphosphate-ribose) polymerase inhibitor
PD-1	programmed death protein 1
PDAC	pancreatic ductal adenocarcinoma
PD-L1	programmed death ligand 1
PFS	progression-free survival
PR	partial response
REP-TILs	TILs expanded in the rapid expansion protocol
SCID	severe combined immune deficiency
SD	stable disease
TAA	tumor-associated antigen
TGF-β	growth factor-β
Th1	T helper type 1-cell
TIGIT	T cell immunoglobulin and ITIM domain
TILs	tumor-infiltrating lymphocytes
TIM-3	T-cell immunoglobulin mucin-3
TLR	toll-like receptor
TMB	tumor mutational burden
TME	tumor microenvironment
TNF-α	tumor necrosis factor-α
TP53	Tumor Protein P53
Treg	regulatory T cells
TSA	tumor-specific antigen
VEGF	vascular endothelial growth factor
WT1	Wilms’Tumor antigen 1
